# Genome-wide analysis of sugar transporter genes in maize (*Zea mays* L.): identification, characterization and their expression profiles during kernel development

**DOI:** 10.7717/peerj.16423

**Published:** 2023-11-17

**Authors:** Nan Sun, Yanfeng Liu, Tao Xu, Xiaoyan Zhou, Heyang Xu, Hongxia Zhang, Renhui Zhan, Limin Wang

**Affiliations:** 1 The Engineering Research Institute of Agriculture and Forestry, Ludong University, Yantai, Shandong, China; 2 Zhaoyuan Shenghui Agricultural Technology Development Co., Ltd., Zhaoyuan, Shandong, China; 3 College of Agriculture, Ludong University, Yantai, Shandong, China; 4 School of Pharmacy, Shandong Technology Innovation Center of Molecular Targeting and Intelligent Diagnosis and Treatment, Binzhou Medical University, Yantai, Shandong, China

**Keywords:** *Zea mays*, Sugar transporters, Phylogenetic analysis, Gene expression, Gene family

## Abstract

Sugar transporters (STs) play a crucial role in the development of maize kernels. However, very limited information about STs in maize is known. In this study, sixty-eight *ZmST* genes were identified from the maize genome and classified into eight major groups based on phylogenetic relationship. Gene structure analysis revealed that members within the same group shared similar exon numbers. Synteny analysis indicated that *ZmSTs* underwent 15 segmental duplication events under purifying selection. Three-dimensional structure of ZmSTs demonstrated the formation of a compact helix bundle composed of 8–13 trans-membrane domains. Various development-related *cis*-acting elements, enriched in promoter regions, were correlated with the transcriptional response of *ZmSTs* during kernel development. Transcriptional expression profiles exhibited expression diversity of various *ZmST* genes in roots, stems, leaves, tassels, cobs, embryos, endosperms and seeds tissues. During kernel development, the expression of 24 *ZmST* genes was significantly upregulated in the early stage of grain filling. This upregulation coincided with the sharply increased grain-filling rate observed in the early stage. Overall, our findings shed light on the characteristics of *ZmST* genes in maize and provide a foundation for further functional studies.

## Introduction

In higher plants, sugars, including monosaccharide and sucrose, play a crucial role in enhancing yield (Büttner, 2007; Julius et al., 2017). In many plant species, sucrose is synthesized in green organs (source) and transported over long distances through the phloem to heterotrophic organs (sink) (Van Bel, 2003). Upon reaching the sink organs, sucrose is either directly transported into sink cells or cleaved into monosaccharides by cell wall-bound invertases, which are subsequently taken up by the sink cells (Sherson et al., 2003). Extensive researches have established that the transport of sugars into sink cells is mediated by sugar transporters (STs), which facilitate the transport of both monosaccharides and sucrose (Noiraud, Maurousset & Lemoine, 2001; Büttner, 2007; Kühn & Grof, 2010).

Many sugar transporters, specifically those from major facilitator superfamily (MFS) and sugar will eventually be exported transporters (SWEET) family, have been identified in various species (Chen et al., 2012; Zheng et al., 2014). MFS is further divided into the monosaccharide transporter (MST) family and the sucrose transporter (SUT) family, with MST family exhibiting greater diversity (Yan, 2013). The MST family members are classified into seven subfamilies, including sugar transporter proteins (STPs) that act as proton/sugar symporters for various monosaccharides (Büttner, 2007), polyol/monosaccharide transporters (PMTs) responsible for transporting monosaccharide and sugar alcohols on the plasma membrane (Noiraud, Maurousset & Lemoine, 2001), sugar facilitator proteins (SFPs) that export hexoses on the vacuolar membrane (Yamada et al., 2010; Klemens et al., 2014), inositol transporters (INTs) that function as H^+^/inositol symporters (Strobl et al., 2018), plastidic glucose translocators (pGlcTs) that export glucose into the cytosol (Cho et al., 2011), and two families of monosaccharide importers for sugar uptake in the tonoplast, namely tonoplast sugar transporters (TSTs) and vacuolar glucose transporters (VGTs) (Aluri & Büttner, 2007; Cheng et al., 2018a; Cheng et al., 2018b). These eight families of MFS-type sugar transporters are ancient and present in both dicotyledonous and monocotyledonous plants (Lemoine, 2000; Johnson, Hill & Thomas, 2006). The SWEET family, discovered in 2010, belongs to another superfamily and possesses seven transmembrane domains (Chen et al., 2010; Xuan et al., 2013). Due to these differences, the SWEET transporter family will not be discussed in this study.

Previous studies have shown the importance of *STs* in the transportation of sugars to sink tissues, which is crucial for crop yield and quality. In *Arabidopsis*, there are 62 identified *AtST* genes. Mutants of *AtSUC2*, which have decreased sucrose transport in the phloem, accumulate excessive starch in the leaves, leading to severe growth inhibition and reduced fertility (Gottwald et al., 2000; Gould et al., 2012). The expression of *AtSTP4* gradually increases during pollen development, with the highest level occurring in mature pollen (Truernit et al., 1996). *AtSTPs* are not been found in the female gametophyte or developing seeds (Büttner, 2010). *AtVGT1*, located on vacuolar membrane, plays an important role in flowering and seed germination by transporting glucose (Aluri & Büttner, 2007). *AtTMT1* and *AtTMT2* transport monosaccharides and sucrose into the vacuole (Schulz et al., 2011). Mutants of *Aterdl6* show increased vacuolar glucose levels and increased seed weight due to higher sugar, protein, and lipid levels (Poschet et al., 2011).

In rice, *OsTMTs* transport glucose into vacuoles and contribute to sugar storage in vacuoles (Cho et al., 2010). *OsSUT1* is involved in long-distance sucrose transport, plant height, pollen vitality and seed germination. Mutants of *OsSUT1* exhibit a slight dwarf phenotype and complete infertility due to failed grain filling (Hu et al., 2021; Sun et al., 2022; Wang et al., 2022). *OspGlcT2* is expressed in response to sugar and salt, indicating its role in salt stress tolerance (Deng et al., 2019). *OsSTP10* is induced by sucrose and fructose treatments in roots, but does not respond to hormone treatments. *OsSTP16* is highly expressed in flag leaf sheaths and responds rapidly to glucose and fructose (Deng et al., 2019). The *OsTMTs* in rice function similarly to *AtTMTs*, transporting monosaccharides into vacuoles (Cho et al., 2010).

Maize (*Zea mays* L.) is a significant global food crop with important economic and social value, as well as applications in the bioenergy industries (Tian et al., 2019). Additionally, it serves as an excellent model organism for genetic and genomic studies due to its high photosynthetic rate, availability of a reference genome and efficient transformation system (Schnable et al., 2009; Wang et al., 2020). *STs* not only play a role in sugar transport and allocation, but also have crucial impacts on plant yield and quality. However, compared to species like strawberry, pear, tomato and rice, limited research has been conducted on the *ST* gene family in maize. In this study, we performed a comprehensive search against the updated maize genome B73_RefGen_v5 and identified 68 *ZmST* genes. Through phylogenetic relationship, chromosome location, collinearity analysis, conservative structures and expression patterns analyses in maize, we found that *ZmSTs* play a significant role in sugar transportation and seed development. These results serve as valuable references for further research on *ZmSTs* and provide new genetic resources for the high-yield maize breeding.

## Materials & Methods

### Plant materials and growth condition

The maize inbred line B73 was used in this study. B73 seeds were sterilized with mercuric chloride and cultured in ddH_2_O at 28 °C (light)/23 °C (dark) with a 16 h light/8 h dark photoperiod (Li et al., 2013). After germination, the seedlings with uniformed growth were selected and moved into the field. Subsequently, various tissues and kernels at different days after pollination were collected for the analysis of *ZmST* expression levels.

### Identification and characterization of ST proteins in maize

To investigate putative *ST* genes in *Zea mays*, two methods were employed. First, 62 AtST sequences in *Arabidopsis* were downloaded and used to perform a BLAST search against the *Zea mays* genome obtained from maizeGDB (https://www.maizegdb.org/) with default parameters (Long et al., 2021). Additionally, the Hidden Markov Model (HMM) profiles of the Sugar_tr domain (PF00083), MFS-1 (PF07690) and MFS-2 (PF13347) were obtained from Pfam (http://pfam.xfam.org/) and utilized for HMMER 3.0 searches against the potential ST proteins in maizeGDB (Prakash et al., 2017; Mistry et al., 2021). All potential ZmST proteins were determined on NCBI (https://www.ncbi.nlm.nih.gov/cdd/) and SMART (https://smart.embl.de/).

### Comparison of the numbers of *ST* gene families in different plants

*ST* genes from various plants, including *Arabidopsis* (Büttner, 2007), rice (Deng et al., 2019), tomato (Reuscher et al., 2014), pear (Li et al., 2015), strawberry (Liu et al., 2020), grape (Afoufa-Bastien et al., 2010), Longan (Fang et al., 2020), and apple (Wei et al., 2014) were analyzed to compare the number of *ST*s across different plant species.

### Chromosomal location, collinearity and duplication event analyses

The chromosomal locations of *ZmST* genes on chromosomes and chromosome synteny were performed by TBtools (Chen et al., 2020). Gene duplication analyses were conducted as previously described. The ratio of non-synonymous substitution rate (Ka) to synonymous substitution rate (Ks) was calculated by TBtools.

### Phylogenetic tree analysis of STs from different plants

Amino acid sequences of STs from *Zea mays*, *Arabidopsis thaliana* and *Oryza sativa* were used to create a phylogenetic tree. The phylogenetic tree was constructed by MEGA 7.0 software using neighbor-joining (NJ) phylogenetic method with 1,000 bootstrap replications (Kumar, Stecher & Tamura, 2016).

### Gene structure, conserved motif and domain analyses

The gene structure of *ZmST* genes was analyzed by TBtools software. The conserved motifs of ZmST proteins were analyzed with MEME (https://meme-suite.org/meme/) (Bailey et al., 2009). The maximum number of predicted motifs was set to 15. The final graph was presented by TBtools.

### Expression heatmap of transcriptome

RNA-Seq datasets from different tissues were acquired from maizeGDB to analyze the expression profiles of the *ZmST* genes (Stelpflug et al., 2015). Ten tissues from maize vegetative development to reproductive development stages were used to identify tissue specificity of *ZmST* genes. The expression data of *ST*s was visualized using the TBtools.

### RNA extraction and qRT-PCR

RNAs from B73 materials were extracted by RNAprep Pure Plant Kit (Tiangen Biotech Co., Ltd., Beijing, China) according to the manufacturer’s instruction. About 1-2 µg of RNA was using to reverse transcribe with HiScript® III All-in-one RT SuperMix reverse kit reagents (Vazyme Biotech Co., Ltd.). In order to eraser gDNA and synthesize cDNA, 4 µl of 5 × All-in-one RT SuperMix, 1 µl of Enzyme Mix, 1–2 µg of RNA and appropriate volume of RNase-free ddH_2_O were mixed. Subsequently, the mixture was incubated at 50 °C for 15 min, followed by a temperature increase to 85 °C for 5 s. The qRT-PCR was performed with primers listed in Table S1, with *ZmACTIN1* as an internal reference. qPCR was run on CFX96TM real-time system (Bio-Rad, Hercules, CA, USA), with ChamQ Universal SYBR qPCR Master Mix (Vazyme Biotech Co., Ltd., Nanjing, China), as previously described (Fang et al., 2023). Finally, the calculation method for *ZmST* genes expression levels was proposed by Livak and Schmittgen (Livak & Schmittgen, 2001).

### *Cis*-acting regulatory elements analysis in the *ZmST* gene promoters

The promoter regions of *ZmSTs* were obtained with TBtools software for promoter analysis. The *cis*-acting regulatory elements were identified by PlantCARE (http://bioinformatics.psb.ugent.be/webtools/plantcare/html/) and presented with TBtools.

## Results

### Sixty-eight *ZmST* genes are identified in maize genome

In this study, a total of 68 gene sequences encoding putative ST proteins were identified. Their physicochemical properties, including gene ID, protein size, molecular weight (MW), isoelectric point (*p* I), the grand averages of hydropathicity (GRAVY), and localization prediction, were characterized (Table 1). The molecular weight of ZmST proteins ranged from 41.83 kDa (ZmSFP1) to 80.96 kDa (ZmTST3), while the isoelectric points ranged from 4.72 (ZmTST3) to 9.82 (ZmSTP12) (Table 1). The grand averages of hydropathicity for all ST proteins indicated their hydrophobic nature. Subcellular localization analysis revealed that all ZmST proteins were located in the cell membrane (Table 1, Table S2).

**Table 1:** Physicochemical characteristics of 68 ST proteins.

**Gene name**	**Gene ID**	**Protein size (aa)**	**MW** ^a^ ** (KDa)**	***p*** **I** ^b^	**GRAVY** ^c^	**Localization prediction**
*ZmINT1*	Zm00001eb425560	509	53.92	5.31	0.609	Cell membrane
*ZmINT2*	Zm00001eb300060	585	62.44	8.82	0.361	Cell membrane
*ZmINT3*	Zm00001eb306230	500	53.18	9.06	0.482	Cell membrane
*ZmINT4*	Zm00001eb426370	591	63.84	8.67	0.364	Cell membrane
*ZmpGlcT1*	Zm00001eb125210	539	56.81	9.22	0.567	Cell membrane
*ZmpGlcT2*	Zm00001eb335350	539	56.59	9.08	0.585	Cell membrane
*ZmpGlcT3*	Zm00001eb311910	550	58.08	6.27	0.466	Cell membrane
*ZmpGlcT4*	Zm00001eb203690	485	52.35	8.59	0.535	Cell membrane
*ZmPMT1*	Zm00001eb008070	556	59.39	8.13	0.335	Cell membrane
*ZmPMT2*	Zm00001eb027550	508	54.03	9.22	0.541	Cell membrane
*ZmPMT3*	Zm00001eb066030	562	58.31	7.14	0.579	Cell membrane
*ZmPMT4*	Zm00001eb075840	534	58.04	6.01	0.431	Cell membrane
*ZmPMT5*	Zm00001eb008080	524	56.15	9.08	0.444	Cell membrane
*ZmPMT6*	Zm00001eb325680	509	54	8.88	0.617	Cell membrane
*ZmPMT7*	Zm00001eb021140	519	55.3	9.53	0.558	Cell membrane
*ZmPMT8*	Zm00001eb107810	516	54.37	9.16	0.621	Cell membrane
*ZmPMT9*	Zm00001eb325640	522	55.59	8.9	0.61	Cell membrane
*ZmPMT10*	Zm00001eb107870	520	55.08	9.16	0.589	Cell membrane
*ZmPMT11*	Zm00001eb325650	513	54.26	9	0.612	Cell membrane
*ZmPMT12*	Zm00001eb411020	489	50.58	8.7	0.599	Cell membrane
*ZmPMT13*	Zm00001eb411000	478	50.16	8.75	0.742	Cell membrane
*ZmPMT14*	Zm00001eb166230	501	52.14	9.25	0.633	Cell membrane
*ZmPMT15*	Zm00001eb166250	487	50.94	8.89	0.658	Cell membrane
*ZmPMT16*	Zm00001eb166210	481	50.2	8.79	0.73	Cell membrane
*ZmSFP1*	Zm00001eb017730	386	41.83	9.03	0.69	Cell membrane
*ZmSFP2*	Zm00001eb017760	510	54.33	6.91	0.547	Cell membrane
*ZmSFP3*	Zm00001eb127290	492	52.06	8.33	0.644	Cell membrane
*ZmSFP4*	Zm00001eb333940	485	51.43	5.67	0.634	Cell membrane
*ZmSFP5*	Zm00001eb344570	506	54.09	9.23	0.635	Cell membrane
*ZmSFP6*	Zm00001eb296190	500	53.74	8.54	0.552	Cell membrane
*ZmSFP7*	Zm00001eb296190	502	54.16	8.31	0.58	Cell membrane
*ZmSFP8*	Zm00001eb296220	642	68.5	9.25	0.476	Cell membrane
*ZmSFP9*	Zm00001eb344010	499	53.28	8.49	0.58	Cell membrane
*ZmSFP10*	Zm00001eb344020	496	52.62	9.08	0.632	Cell membrane
*ZmSFP11*	Zm00001eb344040	499	52.92	9.24	0.613	Cell membrane
*ZmSTP1*	Zm00001eb298310	523	57.04	9.38	0.467	Cell membrane
*ZmSTP2*	Zm00001eb008810	514	56.69	9.05	0.507	Cell membrane
*ZmSTP3*	Zm00001eb000240	525	57.5	9.21	0.515	Cell membrane
*ZmSTP4*	Zm00001eb324180	524	56.91	9.13	0.606	Cell membrane
*ZmSTP5*	Zm00001eb182870	521	56.35	9.21	0.609	Cell membrane
*ZmSTP6*	Zm00001eb171880	514	55.43	7.54	0.647	Cell membrane
*ZmSTP7*	Zm00001eb391140	520	56.83	9.37	0.503	Cell membrane
*ZmSTP8*	Zm00001eb043150	536	56.85	9.35	0.601	Cell membrane
*ZmSTP9*	Zm00001eb207680	521	56.91	9.1	0.603	Cell membrane
*ZmSTP10*	Zm00001eb159990	509	55.65	9.54	0.672	Cell membrane
*ZmSTP11*	Zm00001eb110690	510	55.64	9.43	0.686	Cell membrane
*ZmSTP12*	Zm00001eb377440	518	54.52	9.82	0.581	Cell membrane
*ZmSTP13*	Zm00001eb047630	508	54.32	9.14	0.554	Cell membrane
*ZmSTP14*	Zm00001eb309780	522	57.27	9.41	0.47	Cell membrane
*ZmSTP15*	Zm00001eb098100	518	56.63	9.16	0.527	Cell membrane
*ZmSTP16*	Zm00001eb312640	523	56.2	9.34	0.557	Cell membrane
*ZmSTP17*	Zm00001eb303000	513	56.14	9.04	0.483	Cell membrane
*ZmSTP18*	Zm00001eb244790	522	55.82	9.58	0.58	Cell membrane
*ZmSTP19*	Zm00001eb423910	456	49.86	9.71	0.627	Cell membrane
*ZmSTP20*	Zm00001eb081130	513	54.77	9.12	0.609	Cell membrane
*ZmTST1*	Zm00001eb022230	747	79.55	4.85	0.401	Cell membrane
*ZmTST2*	Zm00001eb239520	745	79.82	5.26	0.397	Cell membrane
*ZmTST3*	Zm00001eb228740	763	80.96	4.72	0.316	Cell membrane
*ZmTST4*	Zm00001eb166700	652	71.7	5.64	0.354	Cell membrane
*ZmVGT1*	Zm00001eb225000	518	55.46	5.72	0.598	Cell membrane
*ZmVGT2*	Zm00001eb211520	559	58.71	9.6	0.624	Cell membrane
*ZmSUT1*	Zm00001eb005460	521	55.17	8.58	0.608	Cell membrane
*ZmSUT2*	Zm00001eb133930	501	53.37	8.84	0.486	Cell membrane
*ZmSUT3*	Zm00001eb048470	508	53.52	7.46	0.584	Cell membrane
*ZmSUT4*	Zm00001eb259340	592	63.14	6.63	0.323	Cell membrane
*ZmSUT5*	Zm00001eb244930	530	56.2	8.63	0.494	Cell membrane
*ZmSUT6*	Zm00001eb183000	530	55.94	8.7	0.554	Cell membrane
*ZmSUT7*	Zm00001eb402200	519	55.09	8.68	0.609	Cell membrane

**Notes.**

a MW, molecular weight.

b *p*I, isoelectric point.

c GRAVY, grand averages of hydropathicity.

### ZmST proteins are divided into eight groups

**Figure 1:**
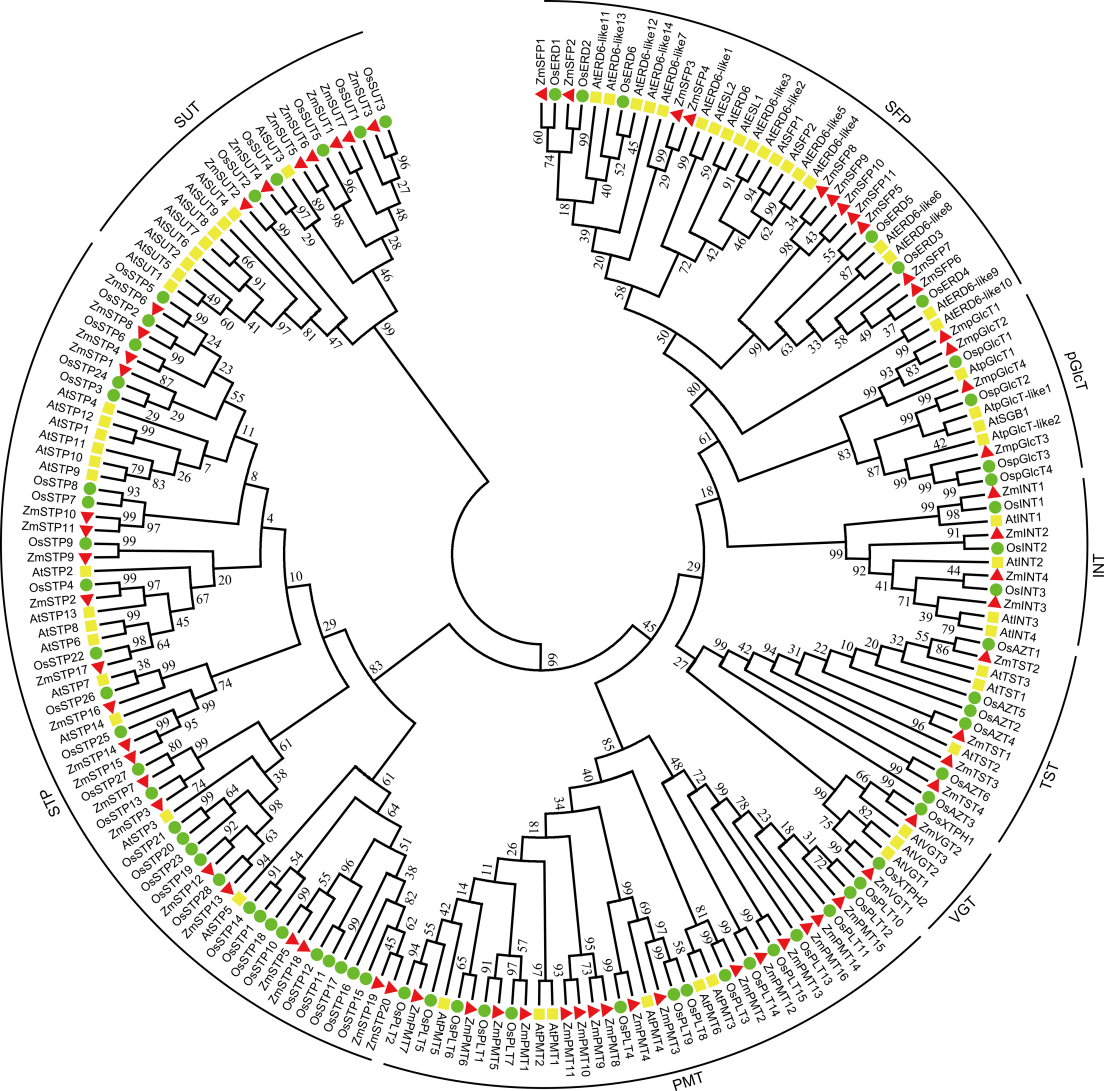
Phylogenetic analysis of sugar transporters from *Z. mays*, *A. thaliana* and *O. sativa*. A total number of 68 ZmSTs from maize, 62 AtSTs from *Arabidopsis* and 69 OsSTs from rice were used to construct the phylogenetic tree by MEGA 7.0 using the neighbor-joining (NJ) method with 1,000 bootstrap replications. All sugar transporter members were classified into eight groups (STP, PMT, SFP, SUT, TST, pGlcT, INT, VGT). The red triangle, yellow square and green circle signs represented *Z. mays*, *A. thaliana* and *O. sativa*, respectively.

A neighbor-joining tree with 199 STs, including 68 ZmSTs from maize, 62 AtSTs from *Arabidopsis*, and 69 OsSTs from rice, was constructed (Fig. 1). The phylogenetic tree suggested that the sugar transporters in maize were classified into eight groups. Among them, the VGT clade consisted of ZmVGT1 and ZmVGT2, while the STP clade contained ZmSTP1 to ZmSTP20. Additionally, the PMT, SFP, SUT, INT, pGlcT and TST clades included 16, 11, 7, 4, 4, and 4 members, respectively, and were annotated as ZmPMT1 to ZmPMT16, ZmSFP1 to ZmSFP11, ZmSUT1 to ZmSUT7, ZmINT1 to ZmINT4, ZmpGlcT1 to ZmpGlcT4, ZmTST1 to ZmTST4 (Fig. 1). Furthermore, phylogenetic analysis showed that there were some closely related orthologous STs between maize and rice, implying the existence of a set of ancestral *ST* genes before the divergence of the two species (Fig. 1).

Additionally, a comparison of the numbers of different *ST* groups among maize, *Arabidopsis*, rice, tomato, pear, woodland strawberry, grape, longan, and apple was carried out (Table 2). The results revealed that STP and PMT were the largest clades in maize, consistent with previous findings in rice, pear, and apple. Conversely, in *Arabidopsis*, tomato, strawberry, grape, and longan, the largest clades were STP and SFP.

**Table 2:** Comparative analysis the gene numbers of different *ST* families in maize, *Arabidopsis*, rice, tomato, pear, strawberry, grape, longan and apple.

**Subfamily**	**Number of genes**								
	**Maize**	***Arabidopsis***	**Rice**	**Tomato**	**Pear**	**Strawberry**	**Grape**	**Longan**	**Apple**
STP	20	14	28	18	20	24	22	20	30
PMT	16	6	15	8	23	7	5	6	10
SFP	11	19	6	10	5	16	22	10	8
SUT	7	9	5	3	6	8	4	6	9
INT	4	4	3	4	6	3	3	4	4
pGlcT	4	4	4	4	6	3	4	3	4
TST	4	3	6	3	6	3	3	1	5
VGT	2	3	2	2	3	2	2	2	3
Total	68	62	69	52	75	66	65	52	73

**Figure 2:**
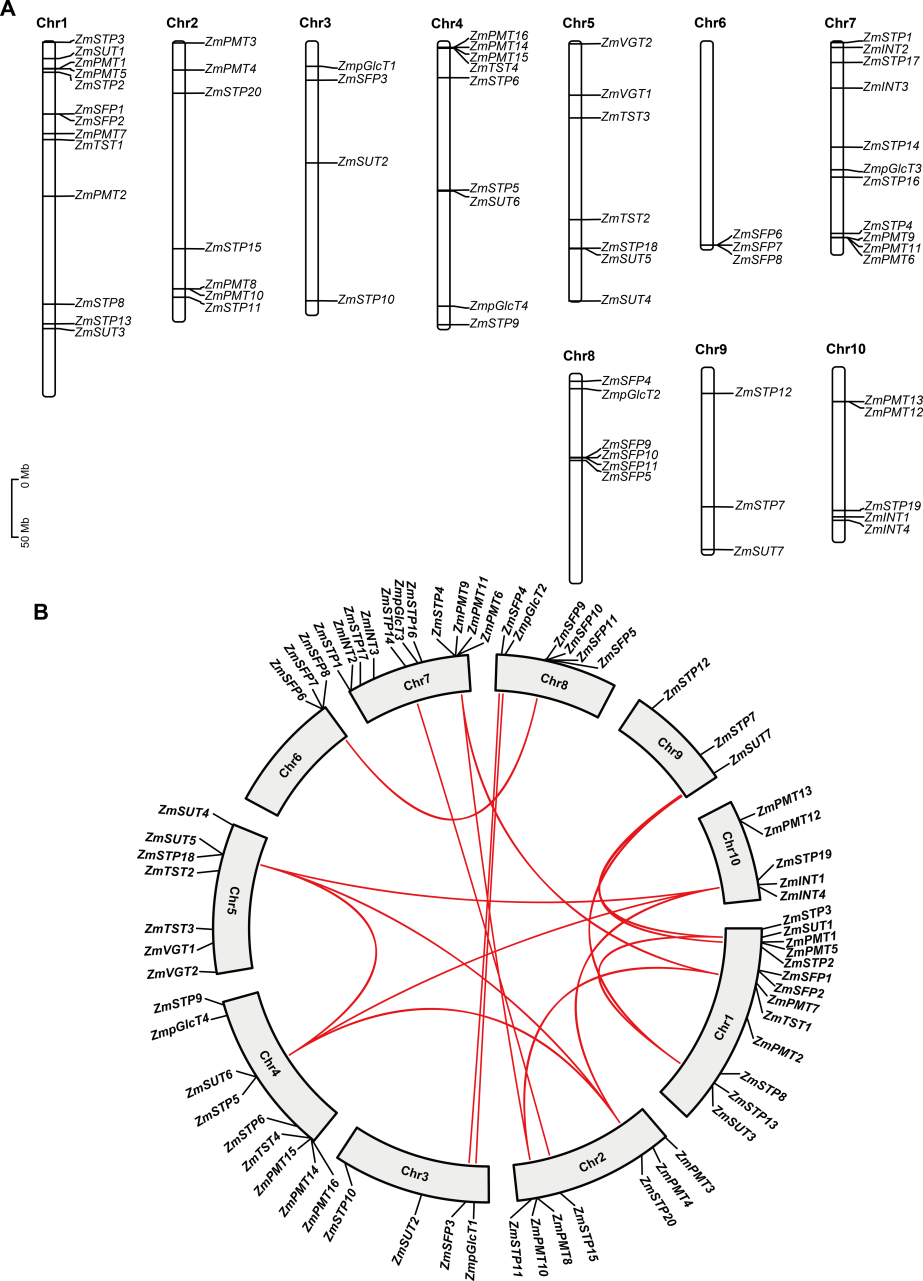
Chromosomal distribution and collinearity analysis of *ZmST* genes. (A) Chromosome distribution of *ZmSTs* in maize genome using TBtools software. The chromosomal location of each *ZmST* gene was mapped according to the maize genome. The chromosome number is indicated at the top of each chromosome. (B) *ZmST* gene duplications analysis with TBtools. The syntenic *ZmST* gene pairs are connected by red lines.

### Segmental duplication events are observed in *ZmSTs*

To investigate features of the *ZmSTs* gene family, we analyzed the chromosome distribution of each *ZmST* gene. Our findings revealed that the *ZmST* genes were located on all 10 chromosomes of maize (Fig. 2A). Chromosome 1 had the highest number of *ZmST* genes, including *ZmPMT1*, *ZmPMT2*, *ZmPMT5*, *ZmPMT7*, *ZmSFP1*, *ZmSFP2*, *ZmSTP2*, *ZmSTP3*, *ZmSTP8*, *ZmSTP13*, *ZmTST1*, *ZmSUT1*, and *ZmSUT3*. Chromosome 2 contained *ZmPMT3*, *ZmPMT4*, *ZmPMT8*, *ZmPMT10*, *ZmSTP11*, *ZmSTP15*, and *ZmSTP20*. *ZmpGlcT1*, *ZmSFP3*, *ZmSTP10*, and *ZmSUT2* were located on chromosome 3. Chromosome 4 harbored *ZmpGlcT4*, *ZmPMT14*, *ZmPMT15*, *ZmPMT16*, *ZmSTP5*, *ZmSTP6*, *ZmSTP9*, *ZmTST4*, and *ZmSUT6*. Chromosome 5 contained *ZmSTP18*, *ZmTST2*, *ZmTST3*, *ZmVGT1*, *ZmVGT2*, *ZmSUT4*, and *ZmSUT5*. Chromosome 6 had *ZmSFP6*, *ZmSFP7*, and *ZmSFP8* genes, while chromosome 7 contained *ZmINT2*, *ZmINT3*, *ZmpGlcT3*, *ZmPMT6*, *ZmPMT9*, *ZmPMT11*, *ZmSTP1*, *ZmSTP4*, *ZmSTP14*, *ZmSTP16*, and *ZmSTP17*. Chromosome 8 harbored *ZmpGlcT2*, *ZmSFP4*, *ZmSFP5*, *ZmSFP9*, *ZmSFP10*, and *ZmSFP11*. Chromosome 9 contained *ZmSTP7*, *ZmSTP12*, and *ZmSU7*, while chromosome 10 harbored *ZmINT1*, *ZmINT4*, *ZmPMT12*, *ZmPMT13*, and *ZmSTP19* (Fig. 2A). Notably, all members of the *ZmVGT* group were located on chromosome 5, and four members of the *ZmINT* group were evenly distributed on chromosomes 7 and 10. Chromosome 6 only contained three *ZmSFP* members. Aside from these observations, the distribution of other *ZmST* genes on maize chromosomes was uneven.

Gene duplications are important for the expansion of gene families (Cannon et al., 2004; Konrad et al., 2011). Collinear analysis showed that 15 gene pairs, *ZmpGlcT1* and *ZmpGlcT2*, *ZmPMT7* and *ZmPMT9*, *ZmPMT7* and *ZmPMT10*, *ZmPMT8* and *ZmPMT9*, *ZmSFP3* and *ZmSFP4*, *ZmSFP6* and *ZmSFP9*, *ZmSTP5* and *ZmSTP18*, *ZmSTP5* and *ZmSTP19*, *ZmSTP5* and *ZmSTP20*, *ZmSTP18* and *ZmSTP19*, *ZmSTP18* and *ZmSTP20*, *ZmSTP19* and *ZmSTP20*, *ZmSUT1* and *ZmSUT3*, *ZmSUT1* and *ZmSUT7*, *ZmSUT3* and *ZmSUT7*, have undergone segmental duplication events (Fig. 2B). Further analysis revealed that all the Ka/Ks values of the *ST* gene pairs were less than 1, indicating that the duplication events occurred under purifying selection (Fig. 2B, Table 3).

### *ZmST* gene structures are highly conserved

**Figure 3:**
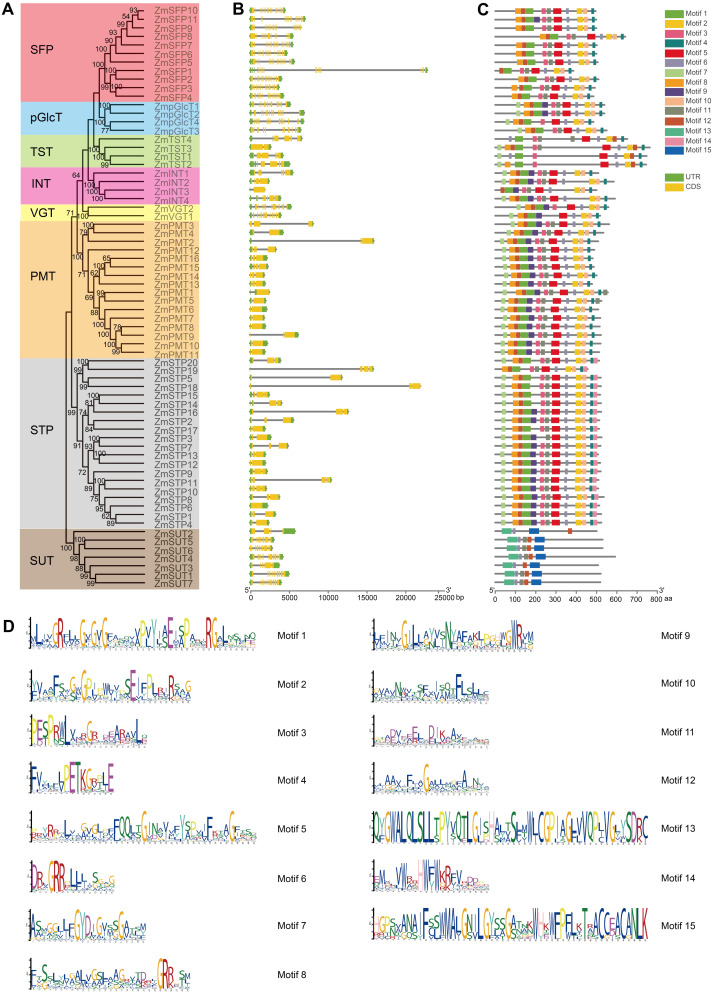
Exon-intron structure and conserved motifs of *ZmST* gene family. (A) Phylogenetic tree of 68 sugar transporters in maize. The phylogenetic tree was constructed by MEGA 7.0 with 1,000 bootstrap replications. Bootstrap values above 50% were considered significant and indicated on the branch nodes. (B) Gene structure analysis of *ZmST* genes. Yellow blocks, black lines and green blocks represented exons, introns and untranslated regions, respectively. (C) The conserved motifs in *ZmSTs*. The different colored boxes represented different motifs. (D) Sequence logos for 15 conserved motifs were performed using MEME online tool. The *x*-axis represented the width of the motif and the *y*-axis represented the bits of each letter.

The gene structures play crucial roles in the evolution and functional diversification of multiple gene families (Lei et al., 2020). The structural analysis of *ZmST*s indicated that all *ZmST* genes, except *ZmSTP6*, harbored at least one intron. Several genes, namely *ZmSFP1*, *ZmSFP2*, *ZmSFP3*, *ZmSFP4*, *ZmSFP5*, *ZmSFP6*, *ZmSFP7*, *ZmSFP8*, *ZmSFP9*, *ZmSFP10*, *ZmSFP11*, *ZmpGlcT1*, *ZmpGlcT2*, *ZmpGlcT3*, *ZmpGlcT4*, *ZmVGT1*, *ZmVGT2*, *ZmSUT1*, *ZmSUT4* and *ZmSUT7* contained more than ten introns. However, genes within the same group usually had a similar number of exons (Figs. 3A and 3B). *ZmSFP1* gene had 19 exons, whereas *ZmSTP6* had only one exon, which indicated that the exons gain and loss occurred during the evolution of *ZmST* gene family. The structures of *ZmSTs* in duplication pairs, such as *ZmpGlcT1* and *ZmpGlcT2*, *ZmPMT7* and *ZmPMT10*, *ZmSFP3* and *ZmSFP4*, *ZmSTP5* and *ZmSTP19*, *ZmSTP5* and *ZmSTP20*, *ZmSUT1* and *ZmSUT7*, were highly similar.

We used the MEME online tool to predict the potentially conserved motifs of 68 ZmSTs. Among the 15 distinct motifs identified, only motif 6 was present in all ZmST proteins (Figs. 3C and 3D, Table S3). Notably, significant differences were observed in the conserved motifs between the SUT subfamily and MST subfamilies, despite their similar function in sugar transport (Figs. 3C and 3D). Motifs 1, 2, 4 and 5 were present in all 61 MST proteins, but were absent in SUT proteins, while motifs 13 and 15 existed in all 7 SUT proteins but not in MST proteins. These findings indicated that motifs 1, 2, 4 and 5 may be critical for the function of MST subfamilies, while motifs 13 and 15 may be necessary for the function of SUT subfamily. This may be due to functional differences between MST subfamilies (monosaccharide transport) and SUT subfamily (sucrose transport). Although all MST proteins contained motifs 1, 2, 4 and 5, the specific types and numbers of motifs varied among each subfamily. Motif 3 was absent in VGT subfamily and ZmSTP3 protein. Motif 7 was not present in any members of the SFP subfamily but was observed in the VGT subfamily. Motif 12 was absent only in ZmINT3 and ZmpGlcT4. Motif 14 was only observed in STP subfamily. Similar motif structures were observed in gene pairs such as *ZmpGlcT1* and *ZmpGlcT2*, *ZmPMT7* and *ZmPMT9*, *ZmPMT7* and *ZmPMT10*, *ZmPMT8* and *ZmPMT9*, *ZmSFP3* and *ZmSFP4*, *ZmSFP6* and *ZmSFP9*, *ZmSTP5* and *ZmSTP18*, *ZmSTP5* and *ZmSTP20*, *ZmSTP18* and *ZmSTP20*, as well as *ZmSUT1* and *ZmSUT7*.

**Table 3:** The Ka/Ks for the duplication gene pairs in *ZmST* family.

**Duplicated pair**	**Duplicate type**	**Ka**	**Ks**	**Ka/Ks**	**Positive selection**
*ZmpGlcT1*/*ZmpGlcT2*	Segmental	0.02302462	0.17639281	0.13053037	No
*ZmPMT7*/*ZmPMT9*	Segmental	0.1887097	0.73309884	0.25741372	No
*ZmPMT7*/*ZmPMT10*	Segmental	0.18220538	0.59309584	0.30721068	No
*ZmPMT8*/*ZmPMT9*	Segmental	0.05922607	0.19262997	0.30746033	No
*ZmSFP3*/*ZmSFP4*	Segmental	0.12261777	0.72591107	0.16891569	No
*ZmSFP6*/*ZmSFP9*	Segmental	0.14390645	0.54307605	0.26498398	No
*ZmSTP5*/*ZmSTP18*	Segmental	0.21542769	0.59259227	0.36353442	No
*ZmSTP5*/*ZmSTP19*	Segmental	0.35185515	0.66335247	0.5304196	No
*ZmSTP5*/*ZmSTP20*	Segmental	0.37342149	0.55823017	0.66893821	No
*ZmSTP18*/*ZmSTP19*	Segmental	0.37160006	0.71051129	0.52300373	No
*ZmSTP18*/*ZmSTP20*	Segmental	0.35668412	0.69017173	0.51680489	No
*ZmSTP19*/*ZmSTP20*	Segmental	0.1612672	0.44168081	0.3651216	No
*ZmSUT3*/*ZmSUT7*	Segmental	0.22699235	0.76180805	0.29796528	No

### ZmST proteins were predicted to form a compact helix bundle

**Figure 4:**
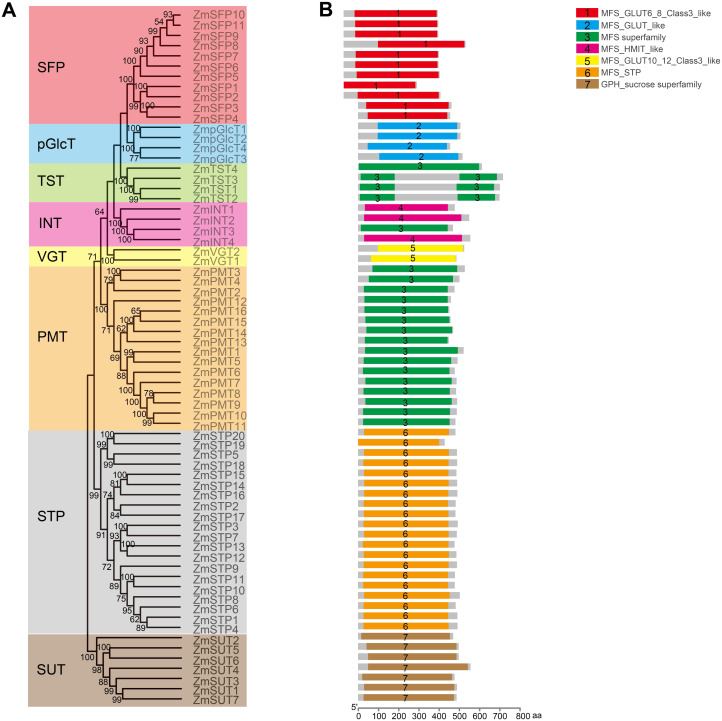
Analysis of the conserved domains in ZmST proteins. (A) Phylogenetic tree of 68 sugar transporters in maize. (B) The conserved domains in ZmSTs were identified with NCBI-CDD. The conserved domains were presented with different colors.

**Figure 5:**
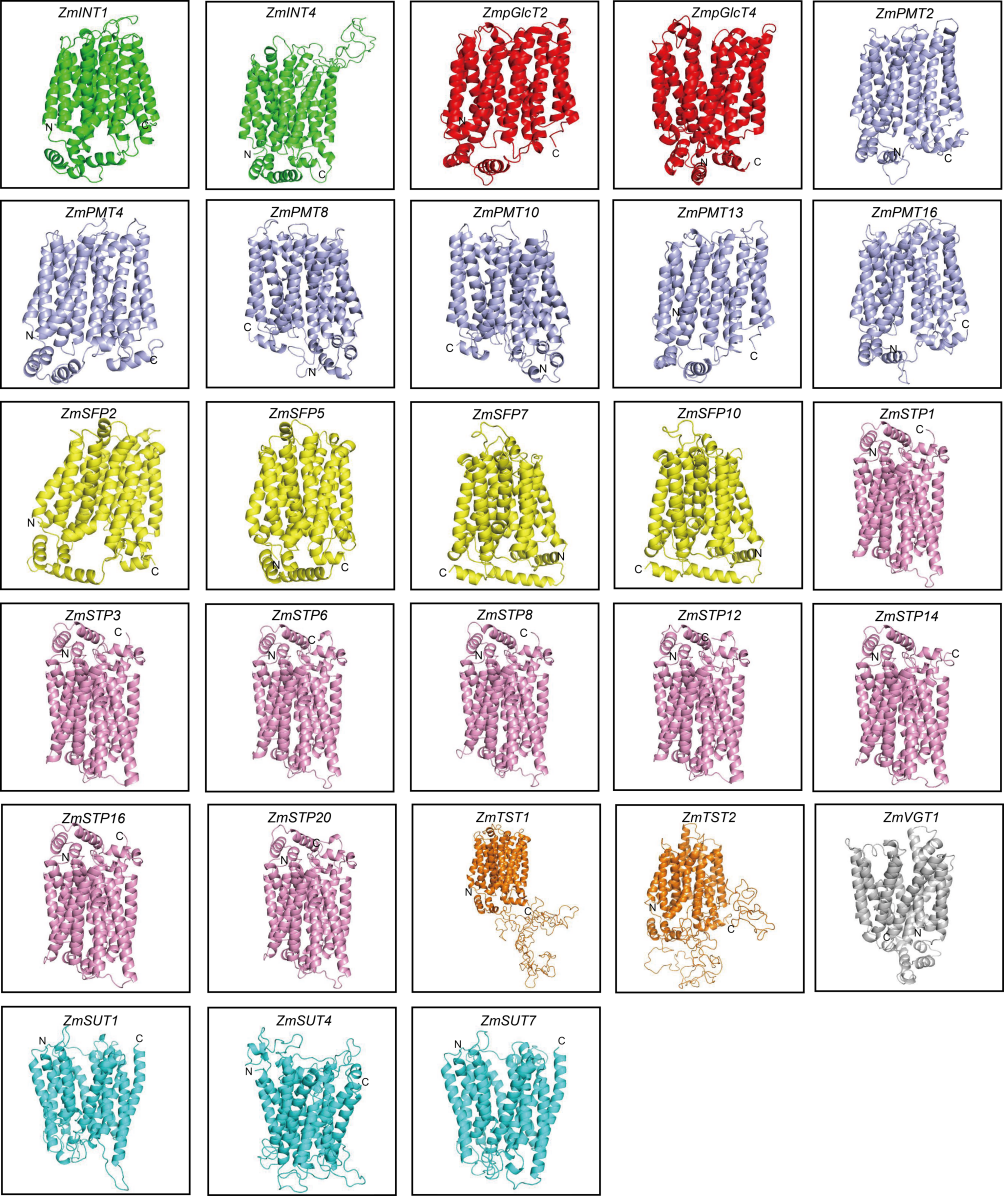
Predicted 3D structures of ZmST proteins. Different subfamilies were represented by different colors. All ZmSTs were folded into 8–13 transmembrane domains to form a compact helix bundle.

In order to explore the potential roles of ZmST proteins, we predicted their conserved domains and 3D models of all ZmSTs with NCBI-CDD and Swiss-model, respectively. All ZmST proteins contained a conserved MFS domain, which facilitated the transportation of various substrates (including sugars, ions, nucleosides, amino acids and so on) through the cytoplasm or inner membrane, except SUT clade. This SUT clade comprised a conserved GPH_sucrose superfamily domain, which might export sucrose from photosynthetic sources to the phloem or import sucrose into sucrose sinks (Fig. 4). Furthermore, 3D prediction demonstrated that all the ZmSTs were folded into 8–13 transmembrane domains, and then formed a compact helix bundle (Figs. 5, S1). However, it’s worth noting that most members within the same group exhibited a similar 3D structure. For instance, the ZmTST subfamily members displayed a unique central loop, composed of approximately 320 amino acids, which connected to the predicted transmembrane domains, a feature absent in all other sugar transporters (Fig. 5).

### *ZmST* promoters contain the *cis*-acting elements for light, phytohormone, stress and development

**Figure 6:**
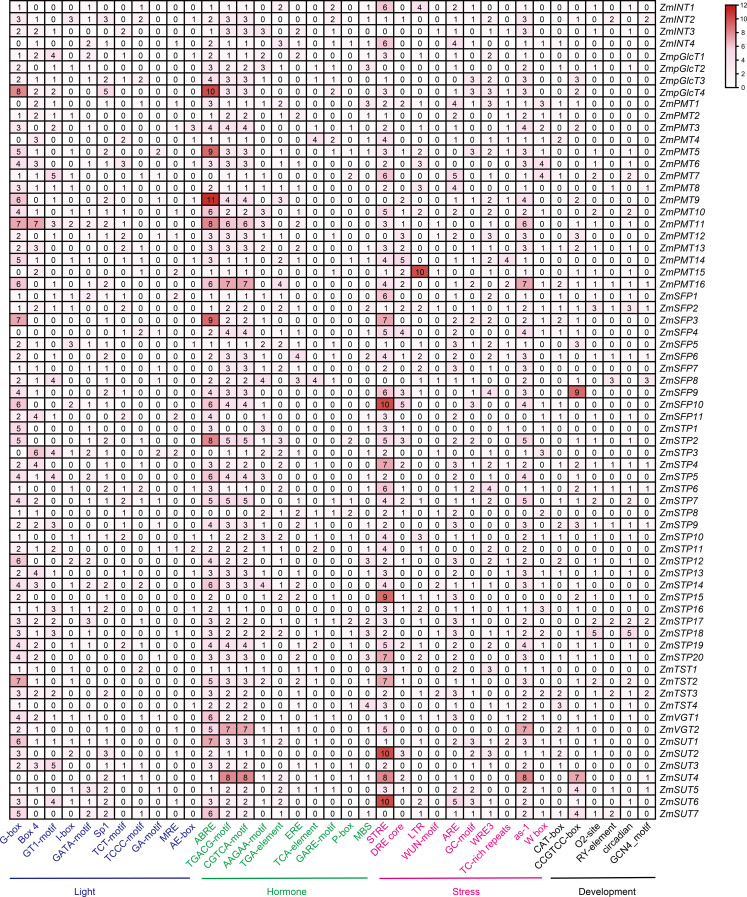
The *cis*-acting elements in the promoters of *ZmST* gene family. The names of 66 *cis*-elements were labeled at the bottom of the figure.

The regulatory *cis*-elements are the binding sites for transcription factors, carrying information to regulate the gene expression in biological pathways. Thus, we extracted the promoter regions of 68 *ZmST* genes and examined their *cis*-acting elements using PlantCARE database 5.0. A total of sixty-six *cis*-elements were identified and categorized into four main groups: photoresponse, hormonal response, stress response and development. Among these *cis*-elements, thirty were related to light-responsive pathway, indicating their role in responding to light, which aligned with the function of sugar transporters in the distribution of photosynthetic products. Additionally, fourteen were associated with hormone response, eleven with abiotic and biotic stress response, and eleven with plant growth and development (Fig. 6). All promoters of *ZmSTs* had the same number of CGTCA-motif and TGACG-motif, and most *ZmSTs* were regulated by abscisic acid and methyl-jasmonate. *ZmSUT4* and *ZmSFP9* had seven and nine CCGTCC-boxes in their promoters, respectively, indicating their potential involvement in meristem-specific activation. The GCN4_motif was present in the promoters of *ZmINT2*, *ZmPMT8*, *ZmPMT16*, *ZmSFP2*, *ZmSFP6*, *ZmSFP8*, *ZmSTP4*, *ZmSTP6*, *ZmSTP9*, *ZmSTP17*, *ZmTST3*, *ZmSUT4* and *ZmSUT5*, suggesting their potential role in endosperm expression. The RY-element was found in the promoters of *ZmINT2*, *ZmPMT8*, *ZmPMT16*, *ZmSFP2*, *ZmSFP6*, *ZmSFP8*, *ZmSTP4*, *ZmSTP6*, *ZmSTP9*, *ZmSTP17*, *ZmTST3*, *ZmSUT5* and *ZmSUT7*, indicating their potential involvement in seed-specific regulation. The most abundant *cis*-elements in *ZmST* promoter regions were G-box, ABRE and STRE, implying that *ZmSTs* may participate in maize’s growth, development and response to light, hormones and stress.

### *ZmSTs* exhibit distinctive expression profiles across ten different tissues in maize

**Figure 7:**
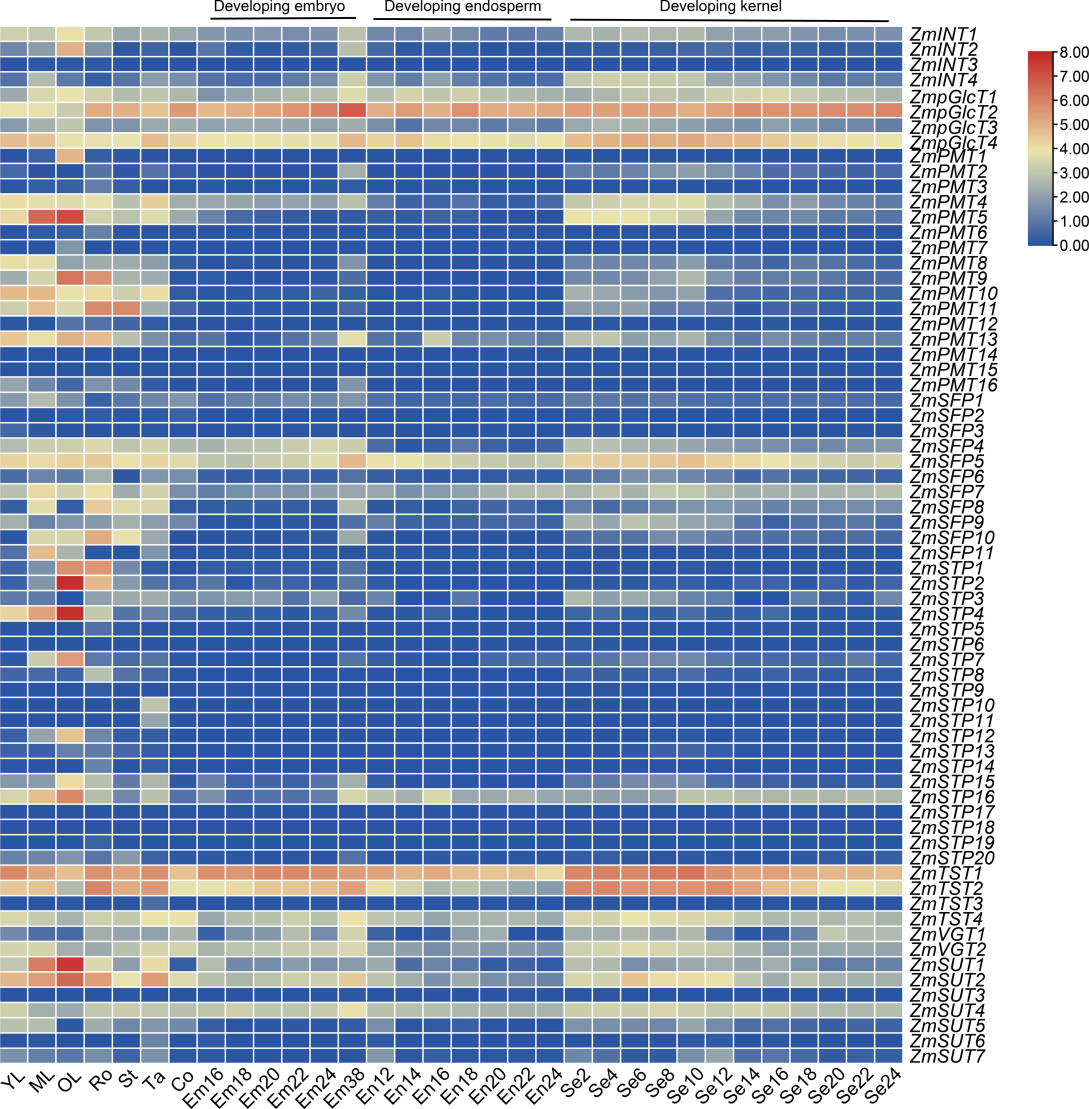
Expression patterns of *ZmSTs* in 10 tissues. The genes were labeled on the right and the tissues were displayed at the bottom of each column. YL, young leaf when the ninth leaf is fully unfolded; ML, mature leaf when the ninth leaf is fully unfolded; OL, old leaf in blister stage; Ro, crown roots node5 when the seventh leaf is fully unfolded; St, stem when the third leaf is fully unfolded; Tassel, miotic tassel when the eighteenth leaf is fully unfolded; Co, immature cob when the eighteenth leaf is fully unfolded; Em16, Em18, Em20, Em22, Em24, Em38: embryo of 16 DAP (days after pollination), 18 DAP, 20 DAP, 22 DAP, 24 DAP, 38 DAP, respectively; En12, En14, En16, En18, En20, En22, En 24: endosperm of 12 DAP, 14 DAP, 16 DAP,18 DAP, 20 DAP, 22 DAP, 24 DAP, respectively; Se2, Se4, Se6, Se8, Se10, Se12, Se14, Se16, Se18, Se20, Se22, Se24: whole seed of 2 DAP, 4 DAP, 6 DAP, 8 DAP, 10 DAP, 12 DAP, 14 DAP, 16 DAP, 18 DAP, 20 DAP, 22 DAP, 24 DAP, respectively.

We downloaded the transcriptome data for *ZmST* genes and generated an expression pattern map using data from ten different tissues: young leaves, mature leaves, old leaves, roots, stems, tassels, cobs, embryos, endosperms, and seeds. Our analysis revealed that nineteen *ZmST* genes (*ZmINT1*, *ZmpGlcT1*, *ZmpGlcT2*, *ZmpGlcT3*, *ZmpGlcT4*, *ZmPMT4*, *ZmPMT13*, *ZmSFP4*, *ZmSFP5*, *ZmSFP7*, *ZmSFP9*, *ZmSTP16*, *ZmTST1*, *ZmTST2*, *ZmTST4*, *ZmVGT1*, *ZmVGT2*, *ZmSUT2* and *ZmSUT4*) showed constitutive expression (log2 (FPKM+1) ≥ 1). Among these genes, *ZmpGlcT1*, *ZmpGlcT2*, *ZmpGlcT4*, *ZmSFP5*, *ZmTST1*, *ZmTST2*, *ZmTST4*, *ZmSUT2* and *ZmSUT4* showed the highest expression levels across all tested tissues and organs (log2(FPKM+1) ≥ 3) (Fig. 7). On the other hand, eleven *ZmST* genes (*ZmINT3*, *ZmPMT14*, *ZmPMT15*, *ZmSFP2*, *ZmSFP3*, *ZmSTP6*, *ZmSTP9*, *ZmSTP17*, *ZmSTP18*, *ZmSTP19* and *ZmSUT3*) showed no expression across all tested tissues and organs (Fig. 7). Furthermore, we observed that two *ZmST* genes (*ZmPMT1* and *ZmPMT7*), four genes (*ZmPMT3*, *ZmPMT6*, *ZmSTP5*, and *ZmSTP14*), and four genes (*ZmSTP10*, *ZmSTP11*, *ZmTST3*, and *ZmSUT6*) exhibited specific expression only in old leaves, roots, and tassels, respectively, with hardly detected expression in other organs. Additionally, we found that *ZmSTP* and *ZmPMT* groups displayed high expression in vegetative organs, whereas *ZmPMT* group members were scarcely detected in developing seeds. However, *ZmTST* group members showed higher transcription level in the developing seeds (Fig. 7).

### Most sugar transporter genes showed a notable upregulation during the early stage of grain filling

**Figure 8:**
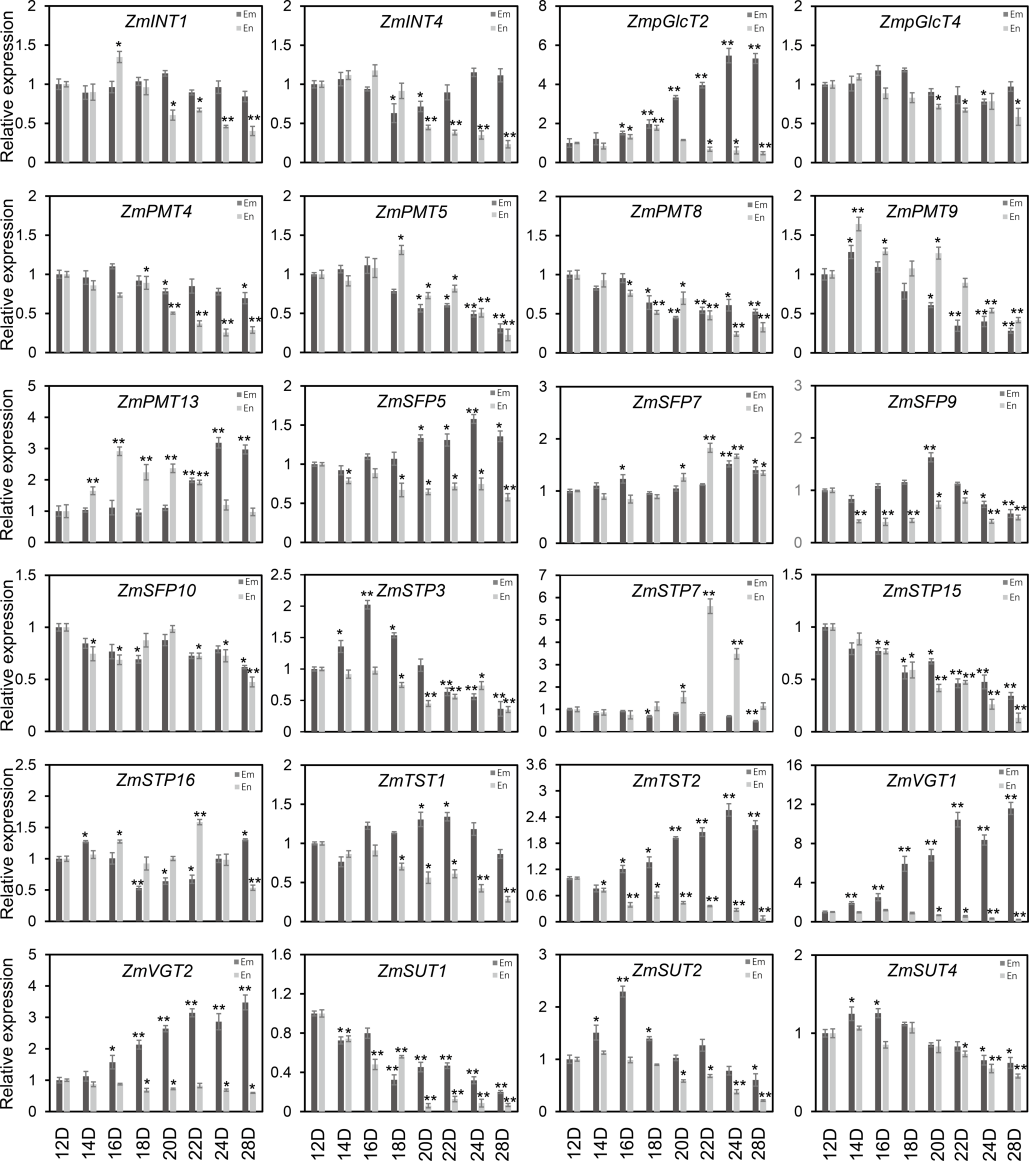
The expression profiles of the *ZmST* genes in embryo and endosperm. The qRT-PCR analysis was used to analyze the expression of selected *ZmST* genes in embryo (Em, shown in dark gray) and endosperm (En, shown in light gray). The names of the genes were labeled at the top of each diagram. 12D, 14D, 16D, 18D, 20D, 22D, 24D, 28D: embryo and endosperm of 12 DAP, 14 DAP, 16 DAP, 18 DAP, 20 DAP, 22 DAP, 24 DAP, 28 DAP, respectively. DAP, days after pollination. Columns were the mean of three independent replicates, and error bars represented SD. * and ** indicated significant differences with *P* < 0.05 and *P* < 0.01, respectively.

**Figure 9:**
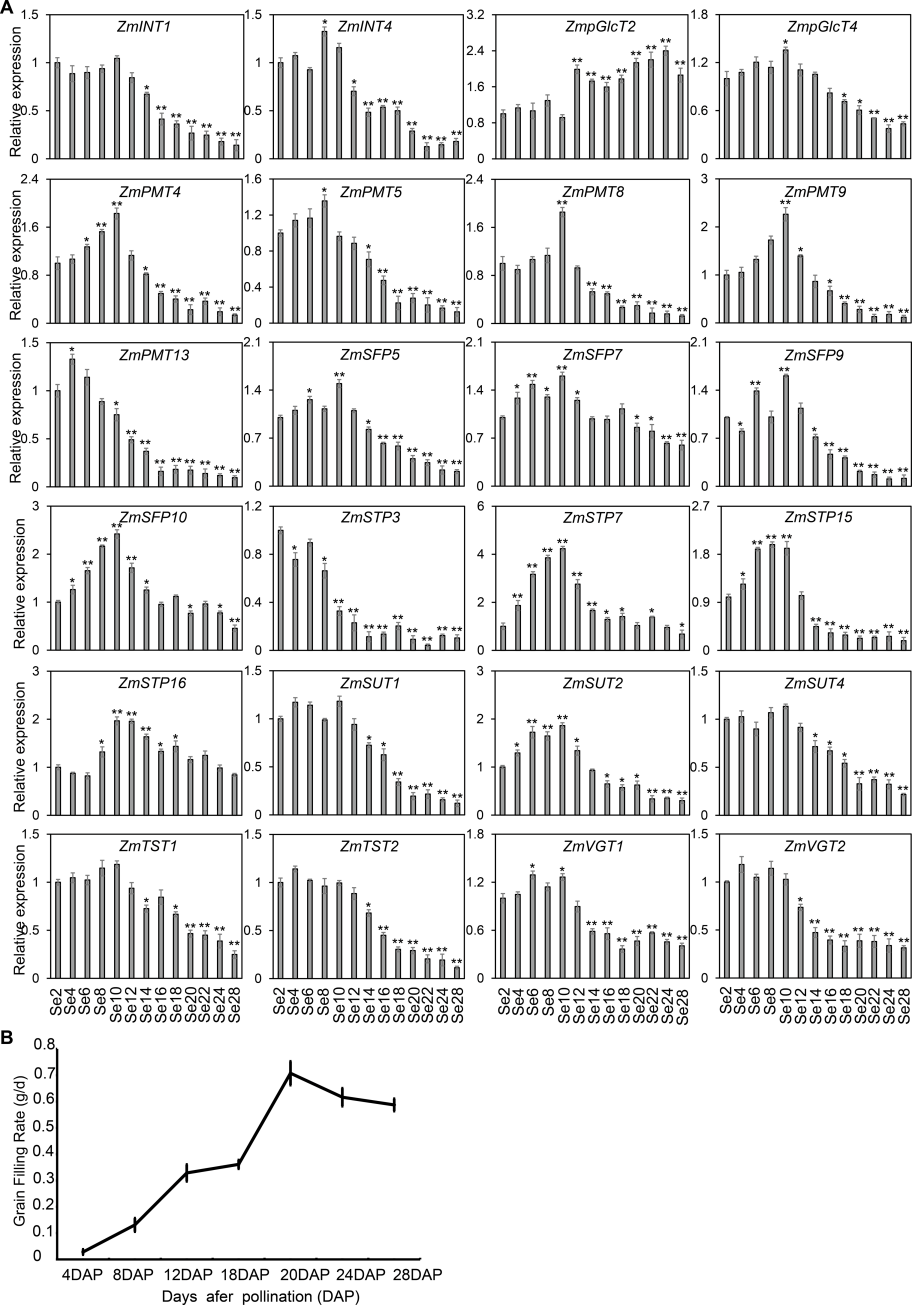
Expression profiles and grain-filling rate of the *ZmST* genes in seed. (A) The qRT-PCR was used to analyze the expression levels of selected *ZmST* genes in seed (Se). The names of the genes were labeled at the top of each diagram. Se2, Se4, Se6, Se8, Se10, Se12, Se14, Se16, Se18, Se20, Se22, Se24, Se28: seed of 2 DAP, 4 DAP, 6 DAP, 8 DAP, 10 DAP, 12 DAP, 14 DAP, 16 DAP, 18 DAP, 20 DAP, 22 DAP, 24 DAP, 28 DAP, respectively. DAP, days after pollination. Columns were the mean of three independent replicates, and error bars represented SD. * and ** indicated significant differences with *P* < 0.05 and *P* < 0.01, respectively. (B) The grain-filling rate calculated by the weight increase of hundred-grain weight was measured from 4 to 28 DAP every 4 days.

As shown in Fig. 7, the diverse expression patterns of *ZmST* genes during stages of embryo, endosperm, and seed development suggested a significant role of *ZmSTs* in maize kernel development. To explore the probable functions of *ZmST* genes, we randomly selected 24 *ZmST* genes representing eight groups and executed qRT-PCR analysis across embryo and endosperm developmental stages as well as seed maturity stages. *ZmpGlcT2*, *ZmSFP5*, *ZmSTP3*, *ZmTST1*, *ZmTST2*, *ZmVGT1*, *ZmVGT2* and *ZmSUT2* showed up-regulation during embryo development and down-regulation during endosperm development. Conversely, *ZmSTP7* were down-regulated during embryo development but up-regulated during endosperm development. Some genes like *ZmINT4*, *ZmPMT4*, *ZmPMT8*, *ZmSFP10*, *ZmSTP15* and *ZmSUT1* were down-regulated, while *ZmSFP7* was up-regulated, during both embryo and endosperm development (Fig. 8). In particular, the expression levels of most tested *ZmST* genes, such as *ZmINT4*, *ZmpGlcT4*, *ZmPMT4*, *ZmPMT5*, *ZmPMT8*, *ZmPMT9*, *ZmPMT13*, *ZmSFP5*, *ZmSFP7*, *ZmSFP9*, *ZmSFP10*, *ZmSTP7*, *ZmSTP15*, *ZmVGT1* and *ZmSUT2*, increased gradually in the early stage of grain filling, reaching a peak at 8-12 days after pollination, and then declined gradually (Fig. 9A). As supported by previous studies emphasizing the significance of the grain filling stage for seed quality and yield (Ji et al., 2022), we investigated the grain-filling rate of B73 over four days from 4 DAP to 28 DAP. Our findings revealed a substantial increase in the grain-filling rate during the developmental stages of 8-12 DAP and 16-20 DAP, aligning with the observed expression pattern of seed maturation (Fig. 9B). These results underlined the crucial role of *ZmST* genes in the embryonic, endospermic, and seed development stages.

## Discussion

The structures, phylogenetic relationship, and functional evolutions of sugar transporters have been extensively studied in various plant species (Julius et al., 2017; Zhu et al., 2021). However, knowledge of their possible roles and regulation processes among different classes of sugar transporters in maize is still limited. In this study, we identified 68 *ZmST* genes in the maize genome and analyzed their physicochemical properties (Table 1). As we know, there were sixty-two *ST* genes in *Arabidopsis* (Büttner, 2007), sixty-five *ST* genes in grape (Afoufa-Bastien et al., 2010), sixty-six *ST* genes in strawberry (Liu et al., 2020), sixty-nine *ST* genes in rice (Deng et al., 2019), seventy-three *ST* genes in apple (Wei et al., 2014), seventy-five *ST* genes in pear (Li et al., 2015). The number of *ST* genes in maize was similar to those found in other plant species, suggesting that the functions of *ST* genes were relatively conserved in functions and crucial for plant growth and development throughout different species. Additionally, the *SWEET* genes, as a newly characterized group of sugar transporters, were identified 20 genes in maize, 23, 22, 24, 21, 29 and 19 genes in sorghum, pearl millet, foxtail millet, rice, barley and *Brachypodium*, respectively (Vinodh Kumar et al., 2023). The number of maize *SWEET* genes was comparable to those observed in other plants. These findings indicated that sugar transporters gene family as well as the *SWEET* gene family in maize, didn’t participate in gene expansion, maintaining the number of genes unchanged.

The subcellular localization of sugar transporters is crucial to explore their potential functions in various biological processes. It is known that sugar transporters are typically localized in membrane system of cells, facilitating the transport of sugar from source leaves to sink tissues, such as developing seeds, providing essential carbon and energy sources for plant growth and development. The sugar content is higher in sink tissues, which requires the proton pumps on the membrane to counteract sugar concentration gradient. The AtSTs in *Arabidopsis* were primarily distributed in the cell membrane and tonoplast (Hedrich, Sauer & Neuhaus, 2015). The *ZmSWEETs* in maize are mainly localized in the plasma membrane, vacuolar membrane, and chloroplast thylakoid membrane (Vinodh Kumar et al., 2023). Consistent with this, ZmST proteins were also mainly located on the cell membrane (Table 1). Based on the phylogenetic relationships in maize, *Arabidopsis* and rice, ZmSTs were clustered into eight groups (Fig. 1), consistent with the previous evolutionary classification of STs from other species. ZmSTs showed a closer relationship with ST proteins from rice, which also belonged to graminaceous crop. This result indicated that the functions of *ST* genes were relatively conserved, especially in graminaceous crops.

In this study, we identified that 68 *ZmST* genes were located on 10 chromosomes in the maize genome (Fig. 2A). In the *ZmST* gene family, we only observed 15 gene pairs with segmental duplication (Fig. 2B, Table 3). Therefore, segmental duplication was probably the predominant driver for the expansion of *ST* gene family in maize. Several duplicated gene pairs, such as *ZmpGlcT1* and *ZmpGlcT2*, *ZmPMT7* and *ZmPMT10*, *ZmSFP3* and *ZmSFP4*, *ZmSTP5* and *ZmSTP20*, *ZmSUT1* and *ZmSUT7*, exhibited similar exon-intron structures and conserved motifs, indicating a certain degree of functional redundancy (Figs. 3B–3D). During the course of evolution, changes in gene structures may lead to variations in expression patterns and functions among *ZmST* genes.

Sugar transporters, divided into MFS-type sugar transporters and SWEET family, are critical for the proper functioning and coordination of various physiological processes during plants growth and development. Previous studies have highlighted the significant roles of MFS-type sugar transporters in different species. *MdERDL6* and *OsTST1* gene play important roles in plant development and kernel maturation in tomato and rice (Zhu et al., 2021; Yang et al., 2023). Zm*SWEETs* showed increased expression levels during maize seed germination, indicating their involvement in nutrient supply to the embryo axis (López-Coria et al., 2019). Moreover, *ZmSWEET* family members exhibited specific expression patterns in embryo and surrounding regions and upregulated expression under abiotic stress (Shen et al., 2022; Vinodh Kumar et al., 2023). So far, there have been many studies reported on sugar transporter proteins in corn, but they are mainly about the SWEET family proteins. However, there are very few reports about the MFS type in maize. Therefore, it is imperative to conduct extensive research on the MFS superfamily in maize to bridge this knowledge gap and gain a comprehensive understanding of their potential roles in maize kernel development. Gene expression patterns can help us understand the biological functions of genes. In *Arabidopsis*, the gene *AtPLT5* was primarily expressed in sink tissues (Reinders, Panshyshyn & Ward, 2005), while in maize, a few members of *ZmPMT* group were expressed in seeds and vegetative organs like old leaves, roots and stems, indicating their involvement in sugar transport to specific sink tissues in plants. Neither *AtSTPs* in *Arabidopsis* nor *ZmSTPs* in maize were found in the female gametophyte or developing seeds (Büttner, 2010), suggesting that the *STP* family may not be involved in the seed maturation process. This suggestion was supported indirectly by previous research, demonstrating the involvement of *ZmSTP2* and *ZmSTP20* in maize disease resistance (Ma et al., 2022). *AtERDL6* orthologs showed higher expression levels in fleshy fruits that accumulated a large amount of sugar, such as tomatoes (McCurdy et al., 2010), oranges (Zheng et al., 2014) and apples (Li et al., 2016). However, *ZmSFPs* in maize showed lower expression levels than fruits, indicating that the role of *SFPs* may not be prominent in crops. *AtSUC2* was expressed in source-leaf companion cells of phloem (Stadler & Sauer, 1996), while *OsSUT1* in rice played a role in sucrose transport during grain filling (Scofield et al., 2002). Similarly, certain *ZmSUTs* showed high expression levels in leaves and developing seeds, implying conserved functions of *SUTs* across *Arabidopsis*, rice and maize. It was reported that the *zmsut2* mutant plant displayed slower growth, smaller ears, and reduced yield compared to WT (Leach et al., 2017). This result implied that the other six ZmSUTs may also play a similar role in seed development. *AtTMT1* gene was strongly expressed in young developing tissues (Wormit et al., 2006), and *ZmTSTs* in maize exhibited higher expression levels in the young leaves, suggesting their involvement in sugar transport during rapid tissue expansion of cells in young tissues. The expression levels of *OsTMT1* and *OsTMT2* in rice were high in various organs excluding the endosperm (Cho et al., 2010), and similarly, *ZmTSTs* in maize were highly expressed in leaves, roots, stems, tassels, embryos and seeds, with lower expression in endosperm. Moreover, *ZmTSTs* showed higher expression levels in the early stage of grain filling compared to the late stage of grain filling, indicating their importance in early seed development. *AtVGT1* was detected in all developmental stages and organs except roots in *Arabidopsis* (Aluri & Büttner, 2007), while *ZmVGT* genes were expressed in roots. Overall, most *ZmPMT*, *ZmSFP* and *ZmSTP* genes were predominantly expressed in vegetative organs and barely expressed in developing kernels, whereas, most *ZmpGlcT*, *ZmVGT* and *ZmTST* genes were highly expressed in seeds (Figs. 7, 8 and 9). Notably, most tested *ZmST* genes exhibited an increase in expression during the early stage of grain filling and a subsequent decline during seed development in maize, suggesting their potential role in grain filling and seed maturation. Overall, our findings deepen our understanding of the pivotal roles of these transporters during maize kernels development and maturation, with the potential to contribute to improving maize yield.

## Conclusions

In summary, sixty-eight *ZmST* genes were identified and systematically analyzed in maize. Gene family analyses were conducted to investigate their physicochemical properties, chromosomal localizations, gene structures and biological functions in maize. The expression pattern analyses suggested that these *ZmST* genes may play a vital role in maize kernel development and could be potential candidates for improving maize yield. These significant findings will serve as valuable references for further research on *ZmSTs* and provide new genetic resources for high-yield maize breeding.

## Supplemental Information

10.7717/peerj.16423/supp-1 Figure S1 Predicted 3D structures of 68 ZmST proteins

10.7717/peerj.16423/supp-2 Table S1 Primer sequences used for qRT-PCR in this study

10.7717/peerj.16423/supp-3 Table S2 Amino acid sequences of ZmST proteins in maize

10.7717/peerj.16423/supp-4 Table S3 Fifteen conserved motifs identified by MEME online tool

10.7717/peerj.16423/supp-5 Supplemental Information 1 MIQE checklist

10.7717/peerj.16423/supp-6 Dataset S1 The raw data for qRT-PCR experiments and the method of calculation

## Additional Information and Declarations

### Data Availability

The following information was supplied regarding data availability: The raw data are available in the Supplementary Files.
