# Supplementary figures and images for: Genome-wide analysis of sugar transporter genes in maize (Zea mays L.): identification, characterization and their expression profiles during kernel development
Source: PeerJ. 2023 Nov 17;11:e16423. doi: 10.7717/peerj.16423 (PMC10658905; doi:10.7717/peerj.16423)

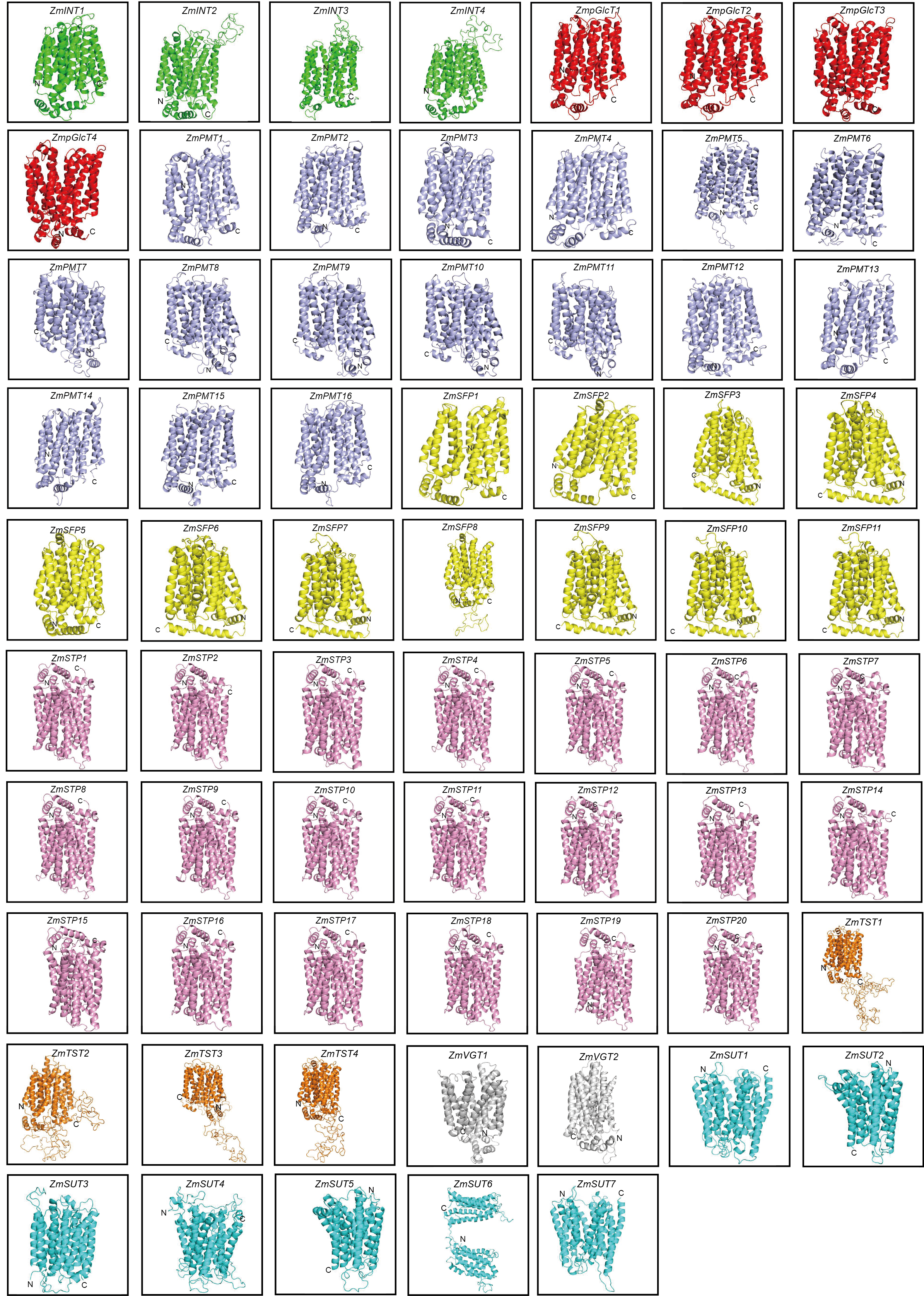

Supplement: Figure S1 [file peerj-11-16423-s001.png]
